# Selectivity of Benzyl Hydroxamic Acid in the Flotation of Ilmenite

**DOI:** 10.3389/fchem.2019.00886

**Published:** 2019-12-24

**Authors:** Lixia Li, Chen Zhang, Zhitao Yuan, Zhichao Liu, Chunfeng Li

**Affiliations:** ^1^School of Resources and Civil Engineering, Northeastern University, Shenyang, China; ^2^Beijing Research Institute of Chemical Engineering and Metallurgy, CNNC, Beijing, China

**Keywords:** ilmenite, benzyl hydroxamic acid, selectivity, density functional theory, flotation

## Abstract

The decreased ground size of ilmenite-bearing ores challenges the selectivity of collectors of ilmenite. Taking advantage of flotation tests and density functional theory (DFT), the selectivity of benzyl hydroxamic acid (BHA) and the adsorption mechanism of oleate and BHA on ilmenite were systematically investigated. The flotation tests showed that BHA had good selectivity to ilmenite. In the DFT study, the favorable adsorption of BHA and oleate on the ilmenite surface were verified by the Mulliken population and the calculated interaction energies. Results indicated that the covalent bonds caused the adsorption of oleate on the ilmenite surface. The strong selectivity of BHA was due to abundant adsorption sites and solid adsorption of five-membered rings. The present investigation has important implications for further studies of BHA and will be helpful for screening and designing collectors for ilmenite flotation.

## Introduction

For the beneficiation of ilmenite-bearing ores, a hybrid beneficiation method of low-intensity magnetic separation (LIMS) for magnetite removal and high-intensity magnetic separation (HIMS) for ilmenite pre-concentration followed by flotation for ilmenite upgrade, is commonly commercialized. With the continuous exploitation of ilmenite-bearing ores in China, the mined resources exhibit lean, fine, and miscellaneous. In order to liberate valuable minerals to a higher degree, fine grinding is applied, which is challenging the selectivity of collectors to ilmenite in the flotation section.

As an anionic collector, benzyl hydroxamic acid (BHA) has been increasingly brought attention to the field of mineral processing in the flotation of tungsten, iron, and rare earth minerals (Bulatovic and Wyslouzil, 1999) owing to its good selectivity. Investigations also showed that BHA as a collector can effectively separate ilmenite from associated gangue minerals, especially fine particles of ilmenite disseminated in the gangue minerals (Belardi et al., 1998; Li et al., 2016).

Many researchers have used hydroxamic acid as a collector to separate different minerals by flotation. Ren et al. (1997) used naphthyl hydroxamic acid for the flotation of bastnaesite. The flotation results showed that the naphthyl hydroxamic acid had good selectivity on the bastnaesite with regards to chemisorption. Sreenivas and Padmanabhan (2002) studied the floatability and adsorption characteristics of BHA on cassiterite. The results showed that the adsorption capacity of cassiterite for octyl hydroxamic acid was greatest under acidic conditions. The optimal slurry pH also changed greatly as the carbon chain of hydroxamic acid increased and the cassiterite particle size decreased. Beyond that, the cyclohexyl hydroxamic acid (Zhao et al., 2013) and octyl hydroxamic (Meng et al., 2015) were employed in the flotation of scheelite flotation and fine wolframite flotation. Novel carboxyl hydroxamic acids (Jiang et al., 2010) were also be designed and used for the flotation of aluminosilicate minerals. In the field of titanium minerals flotation, relevant studies have used hydroxamic acid as a collector (Buckley and Parker, 2013; Meng et al., 2015; Somasundaran, 2018; Chen et al., 2019). It can be seen that various forms of hydroxamic acid are attracting interest in mineral processing due to their unique properties.

During the flotation process, collectors can anchor on the mineral surface as a medium between mineral and air bubbles (Rao and Forssberg, 1991). In the present flotation of ilmenite ore, fatty acids and their modified products commonly serve as collectors industrially. BHA has been proving as a potential alternative to fatty acids due to its good selectivity to ilmenite (Bulatovic and Wyslouzil, 1999), and experiments demonstrate that the flotation performance of BHA is significantly better than that of oleate (shown in section Flotation Results). In view of this phenomenon, based on the mechanism of the collector, two hypotheses have been advanced: that BHA has more adsorption sites on ilmenite surfaces than that of oleate; and that the bond between BHA and the ilmenite surface is more solid than for oleate. Density functional theory (DFT) was applied to energy calculation and bond analysis to provide a quantitative comparison (Li et al., 2019).

DFT is able to reveal the reaction mechanisms that are difficult to explain by current experimental techniques at the atomic level (Pradip et al., 2002). It has been widely used (Kwon and Kubicki, 2004; Blanchard et al., 2012) to investigate properties of molecules and minerals surface as well as the interaction of adsorbent on the adsorbate. The properties of molecular, interaction energies, and Mulliken population analysis give a vivid characterization to the sorption mechanism and provide deep insight into the separation process from the aspects of physical and chemical (Rath et al., 2014). In our previous study, the mechanism of depression of hematite and the adsorption mechanism of oleate on siderite were investigated using DFT (Pradip et al., 2002; Li et al., 2018, 2019; Zhang et al., 2019, 2020).

The aim of this paper was to identify the adsorption configuration of BHA and oleate on the surface of ilmenite, using flotation tests to testify the selectivity of oleate and BHA, and applying DFT to demonstrate the adsorption mechanism. First, flotation tests were conducted at optimal conditions for BHA and sodium oleate, respectively, and the grade and recovery of ilmenite concentrate were compared. Second, the exposed surface of the ilmenite crystal was determined based on the calculation of surface energy. Finally, different models of oleate and BHA were built and screened. The models with the lowest adsorption energy were calculated to analyze the mechanism of adsorption.

## Materials and Methodology

### Experimental Materials

The fine ilmenite sample involved in this study was a classifier overflow materials in a titanium beneficiation plant of Panzhihua Iron & Steel Group Co. in China, with a particle size of −0.038 mm passing 86.18% and TiO_2_ grade of 8.89%. After treated by a LIMS and a superconducting magnetic separator, it was upgraded to 17.01% of TiO_2_. This concentrate was collected, dried, homogenized, and sampled to perform flotation experiments.

BHA and sodium oleate were used as collectors. In order to prepare the solution of sodium oleate, 50 mg of sodium oleate was put into a 500 mL beaker placed in an ultrasonic dispersion machine. Then the dispersed solution was transferred into a 1 L volumetric flask with deionized water to a final concentration of 50 mg/L. The same procedure was followed to prepare a BHA solution with a concentration of 50 mg/L. All experiments were carried out at room temperature. Lead nitrate Pb(NO_3_)_2_ had an analytical purity and was adopted as an activator, and the frother terpenic oil was commercially pure.

### Flotation Experiments

An XFG flotation machine with 1 L flotation cell was used for the flotation experiments with a stirring rate of 1,800 rpm. With BHA and sodium oleate as a collector separately, experimental optimizations with parameters of slurry pH, dosages of reagents, and flotation time, have been implemented. In each experiment, 300 g of samples were added in 700 mL of water to stir for 3 min. Sulfuric acid (H_2_SO_4_) or hydroxide sodium (NaOH) was employed to adjust slurry pH from 4 to 12.

In the case of BHA, after pH adjustment, activator, collector, and frother were added in sequence and conditioned for 3, 5, and 1 min correspondingly. After that, the flotation test was carried out, and the froth was scrapped off every 10 s. While in the case of sodium oleate, collector, and frother were added into the slurry after pH adjustment at the same time intervals to those of BHA. When flotation completed, both floated and sunk fractions were dried in an oven at 60°C, and then weighed and chemically analyzed.

As a result, suitable flotation conditions with BHA for ilmenite were determined as pH of 7.0, PbNO_3_ of 200 g/t, BHA of 1,300g/t, frother of 60 g/t and flotation time of 5 min. While for sodium oleate, optimized flotation conditions were pH of 4.5, sodium oleate of 1,500g/t, 40 g/t of frother and flotation time of 5 min.

### Models for Ilmenite Crystal

The crystal optimization convergence tests of exchange-correlation potentials, k-point, and cutoff energy were carried out to get the optimum ilmenite crystal geometry. The exchange-correlation potentials of generalized gradient approximation (GGA) convergence test included GGA-RPBE, GGA-WC, GGA-PBE, GGA-PBESOL, and GGA-PW91. The convergence criteria of max. displacement = 0.002 Å, energy = 2.0 × 10^−5^ eV, max. stress = 0.1 GPa, and max. force = 0.05 eV/Å. The lattice parameters of ilmenite from the literature and experimental values were referred to ensure the suitability of optimized ilmenite crystal (Wechsler and Prewitt, 1984). In this study, the CASTEP module in Materials Studio was employed for all calculations.

**Figure 1:**
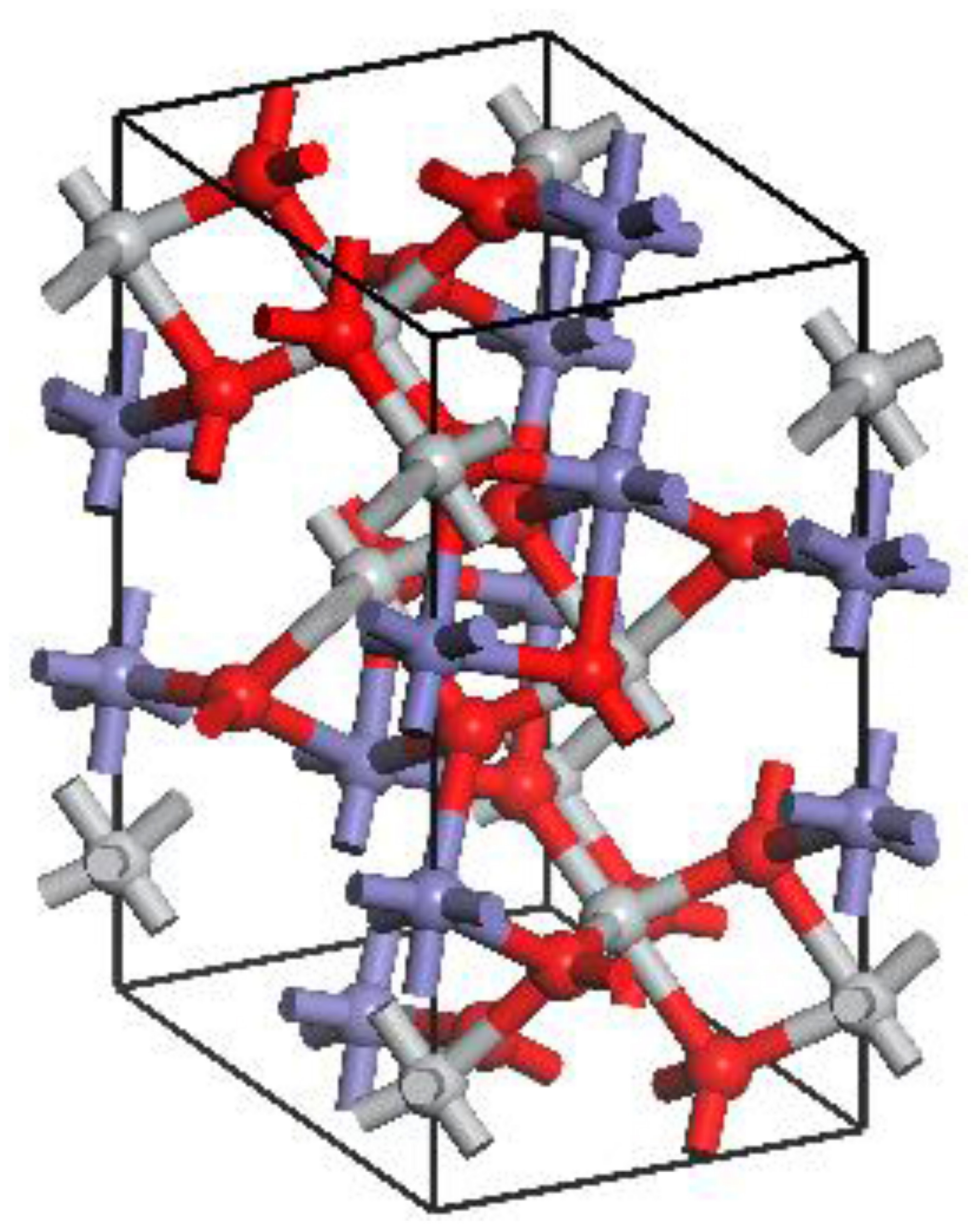
Optimum ilmenite crystal. The red spheres represent O atoms, the gray represents Ti atoms, and the blue represents Fe atoms.

Based on the results of these convergence tests, the cutoff energy of 300 eV, exchange-correlation potential of GGA-PW91 and k-point set 6 × 6 × 2 were selected with the energy −22717.6637 eV and the parameter difference 1.4%, which confirmed the suitability of parameter selection for accurate calculations. Figure 1 shows the optimum ilmenite crystal.

### Models for the Ilmenite Surface

To get the most likely exposed crystal surface of ilmenite, the surface energy calculation was conducted. Surface energy was a thermodynamic stability measurement of an exposed crystalline surface. The smaller surface energy corresponds to a more stable surface structure (Lavina et al., 2009). In this study, the Equation (1) was used to calculate the surface energy (Hu et al., 2012).

(1) $$\begin{array}{r} E_{surf}=\frac{E_{slab}-\left(\frac{N_{slab}}{N_{bulk}}\right)\cdot E_{bulk}}{2A} \end{array}$$

where *E*_*bulk*_ and *E*_*slab*_ are the bulk unit cell and the total energy of the slab, respectively; *N*_*bulk*_ is the number contained in each bulk unit cell and *N*_*slab*_ is the number of atoms in ilmenite slab; unit area of the surface was represented by *A* and the factor of 2 represents the two surfaces in the surface slab that are perpendicular to the z-axis.

In this study, the ilmenite crystal surfaces (1 0 4), (1 1 0), (1 1 −6), (0 1 2), (0 2 4), and (3 0 0) were selected for comparison with the most readily existing crystal surfaces, according to the XRD results. The selected crystal surface was then used to establish the model of ilmenite with reagents.

### Models of Reagents

The representative of modeled conditions to experimental flotation conditions has been considered. Slurry pH determines the predominant functional groups of reagents. As abovementioned, with sodium oleate as a collector, the optimized flotation pH for ilmenite samples was 4.5, while for BHA it was 7.0. Well-known that the predominant group for sodium oleate at pH of 4.5 is oleate molecule, for BHA and Pb(NO_3_)_2_ at pH of 7.0, BHA molecule and Pb(OH)^+^ are dominant species.

In this study, the surface of ilmenite was the adsorbent and the BHA, oleate, and Pb(OH)^+^ were adsorbates. The Visualizer module was employed to design the oleate, BHA and Pb(OH)^+^ molecular structures. Before adsorption modeling, geometric optimizations of the oleate, BHA and Pb(OH)^+^ molecular structures were conducted using the CASTEP module.

## Results and Discussion

### Flotation Results

Using BHA and oleate as collectors, flotation tests were performed at their optimal conditions. Flotation performances in terms of grade and recovery of ilmenite are presented in Table 1.

**Table 1:** Flotation results of BHA and oleate.

**Collector**	**Product**	**Yield/%**	**TiO_**2**_ grade/%**	**TiO_**2**_ recovery/%**
BHA	Concentrate	16.38	39.25	37.79
	Tailings	83.62	12.65	62.21
	Feed	100.00	17.01	100.00
Oleate	Concentrate	32.95	28.42	55.06
	Tailings	67.05	11.40	44.94
	Feed	100.00	17.01	100.00

As can be seen from Table 1, BHA enriched the TiO_2_ grade from 17.01% of the feed to 39.25% of the concentrate which was higher than 28.42% obtained with oleate. In contrast, BHA displayed relatively poor floatability for ilmenite, i.e., with oleate as collector the recovery of TiO_2_ in concentrate was 55.06%, which was higher than that of 37.79% with BHA. The results show that BHA has good selectivity to ilmenite and oleate has good floatability to ilmenite.

### Selected Ilmenite Surfaces

The dominant crystal face of ilmenite was determined by surface energy. The crystal morphology is dominated by the crystal surfaces with a slow-growing rate, meanwhile, a slower growth rate means that a surface has lower surface energy, while fast-growing faces with higher surface energy may disappear (Prywer, 2001). Ilmenite surfaces (1 0 4), (1 1 0), (1 1 −6), (0 1 2), (0 2 4), and (3 0 0) were modeled as a six-layer slab with a vacuum thickness of 25 Å, and the most stable crystal surfaces were determined based on the calculated surface energies as shown in Table 2.

**Table 2:** Surface energies of different ilmenite surfaces.

**Crystal surface**	***E_***bulk***_*/eV**	***E_***slab***_*/eV**	***A*/Å^**2**^**	***E_***surf***_*/J·m^**−2**^**
(1 0 4)	−22717.650	−22711.667	94.25	0.509
(1 1 0)	−22717.650	−22704.376	92.57	1.149

The calculation process for surfaces (1 1 −6), (0 1 2), (0 2 4), and (3 0 0) did not converge, which indicated that the types of atom exposed on the crystal surfaces were unlikely to exist, while the surfaces (1 0 4) with surface energy of 0.509 J·m^−2^ and (1 1 0) with 1.149 J·m^−2^ converged to a stable surface slab. The surface energy of (1 0 4) was lower than that of (1 1 0), which implied that the surface (1 0 4) would more likely exist. Therefore, the surface (1 0 4) was adopted in the subsequent calculations to establish the mineral-reagent models.

### Models for Mineral-Reagent Complex

Models of oleate and BHA on ilmenite surface were built and calculated to reveal the greater selectivity of BHA as compared with oleate. Bond strength was revealed by the adsorption energy and Mulliken bonds population. The adsorption energy (Δ*E*_*ads*_) of reagents and optimized ilmenite (1 0 4) were quantified by Equation (2) (Hu et al., 2012):

(2) $$\begin{array}{r} \Delta E_{ads}=E_{complex}-\left(E_{adsorbate}+E_{mineral}\right) \end{array}$$

where *E*_*complex*_ is the energy of optimized ilmenite-reagent complex, *E*_*adsorbate*_ refers to the total energy of the reagents and *E*_*mineral*_ is the total energy of the ilmenite crystal surface. A greater (negative) magnitude of Δ*E* implies a stronger interaction between the mineral surface and reagent.

Mulliken population analysis was conducted to specify the bond types between adsorbent and adsorbate. The Mulliken bond population is positive for a covalent bond, and the covalent bonding becomes stronger with the increase of Mulliken population. Similarly, a negative bond population indicates antibond.

#### Models for Ilmenite-Oleate Complex

**Figure 2:**
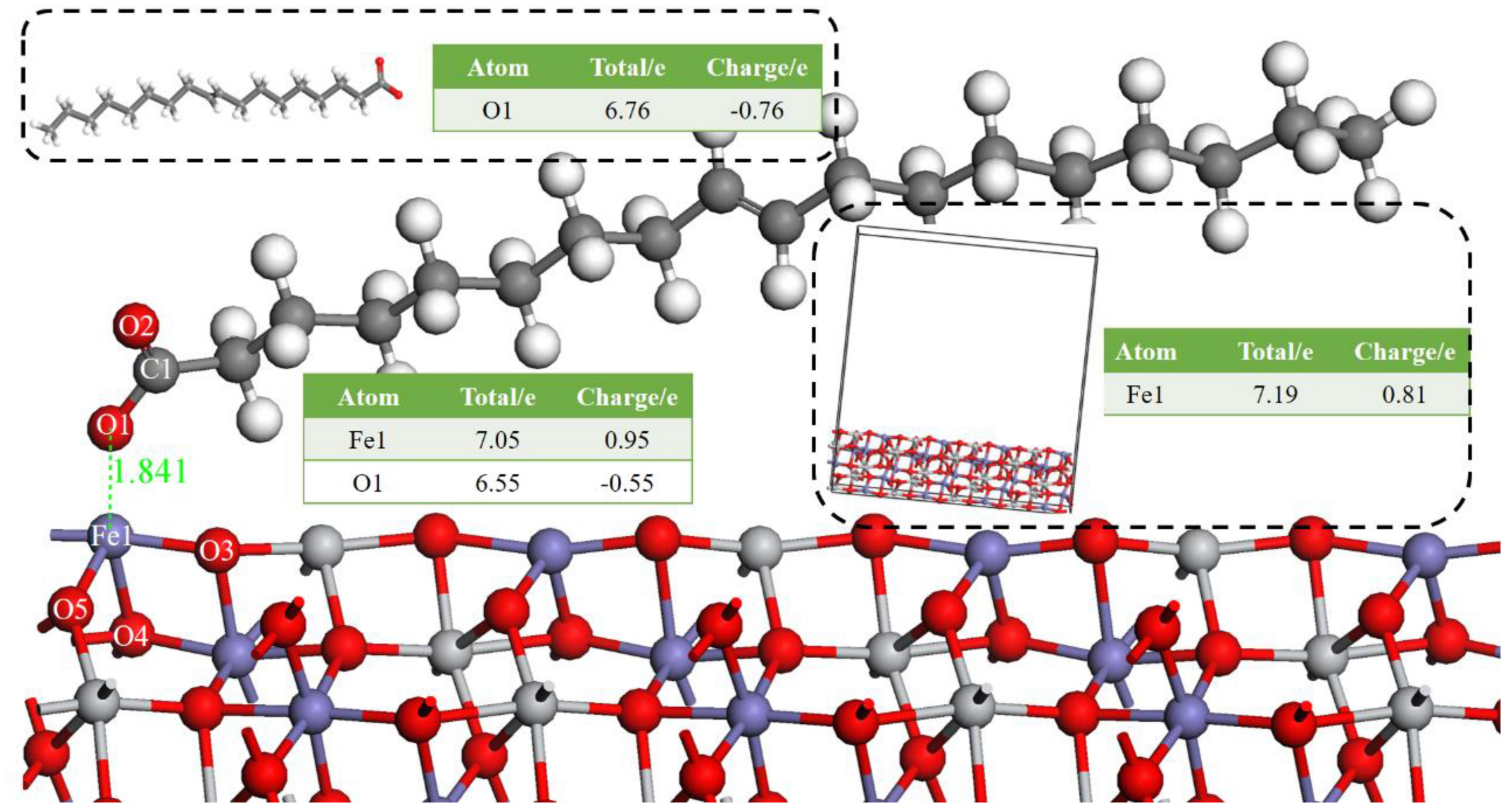
The optimal bond of oleate on an ilmenite (1 0 4) surface, the red ball is O, the blue ball is Fe, the light gray ball is Ti, the dark gray ball is C, and the white ball is H.

Figure 2 shows the apparent interaction of oleate with the atoms on ilmenite surface. The adsorption energy (Equation 2) of oleate on the surface of ilmenite was −146.501kJ/mol, which demonstrated that the oleate adsorbed on the ilmenite surface with a distance of 1.841Å between the Fe1 and O1 atoms. Mulliken population analysis was conducted to discern the bond type of oleate on ilmenite. The bond populations are listed in Table 3.

**Table 3:** Mulliken population of adsorption configuration of oleate on ilmenite.

**Atom**	**Bond**	**Bond population**	**Bond length/Å**
Fe1	O1-Fe1	0.42	1.840
	O5-Fe1	0.25	1.880
	O3-Fe1	0.31	1.893
O1	O3-O1	−0.01	2.830
	C1-O1	0.74	1.340

As shown in Table 3, the bond population of O1-Fe1 was 0.42, indicating that a covalent bond was generated between the Fe1 on the ilmenite surface and O1 of oleate. The bond population of O1-Fe1 was lower than that of C1-O1 in oleate (0.74), yet higher than O5-Fe1 (0.25) and O3-Fe1 (0.31) in the ilmenite crystal, indicating that the interaction between the atoms of Fe and O was strong enough to trigger the adsorption of oleate on the ilmenite surface. The atom population in Figure 2 demonstrates that the charge of O1 and Fe1 are −0.76 e and 0.81 e before the adsorption of oleate on ilmenite surface. After a series reaction, the oleate absorbed on the surface of ilmenite. The total charge of O1 changed from −0.76 e to −0.55 e with 0.21 e lose of charge. Meanwhile, the charge of Fe1 went from 0.81 e to 0.95 e, which manifests the electron transfer existed between O1 and Fe1 atom, which contributed to the formation of covalent bonds.

#### Models for Ilmenite—Pb(OH)^+^—BHA Complex

**Figure 3:**
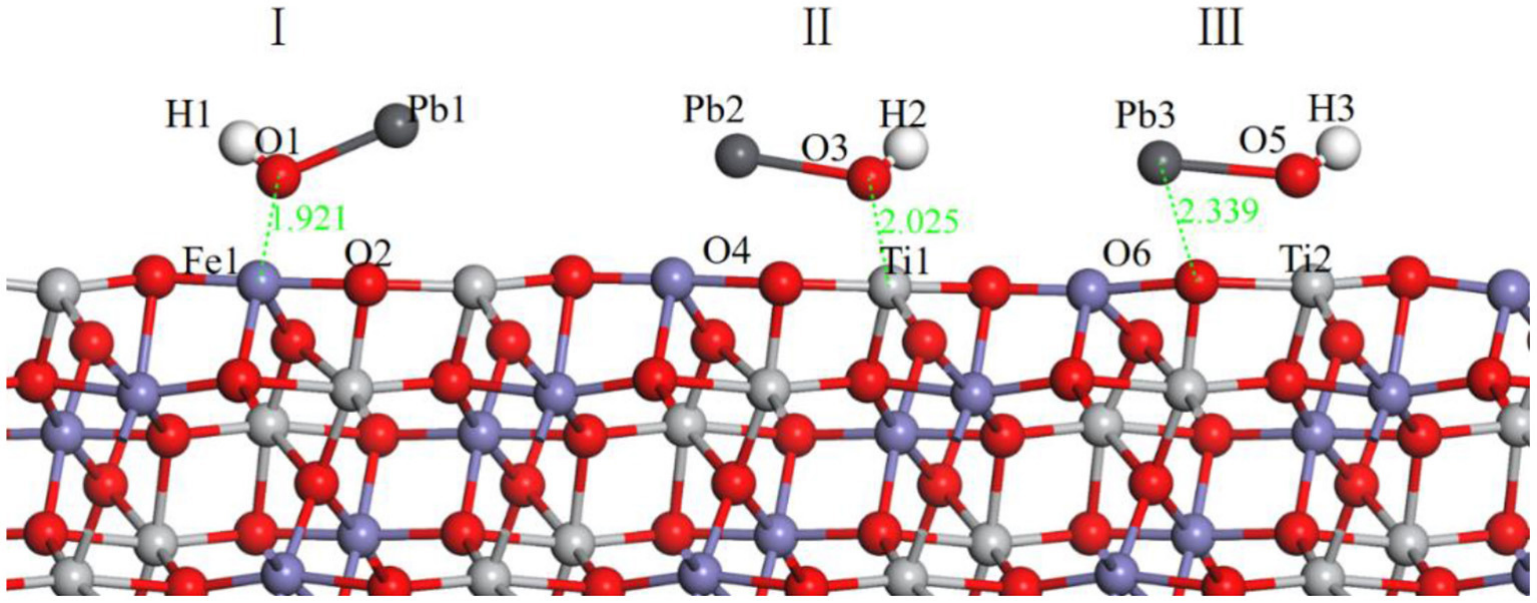
Models of Pb(OH)^+^ on ilmenite surface, (I) represents the Pb(OH)^+^ on the top of Fe atom, (II) represents the Pb(OH)^+^ on the top of Ti atom, (III) represents the Pb(OH)^+^ on the top of O atom. The black ball is Pb.

In the flotation of ilmenite with BHA, the activator lead nitrate is usually added before the collector BHA. Therefore, the adsorption of Pb(OH)^+^ on the ilmenite surface was calculated first. The different models of Pb(OH)^+^ are shown in Figure 3.

During building the models of Pb(OH)^+^ on the ilmenite surface, Pb(OH)^+^ was placed on the top of Fe, Ti, and O atoms of the ilmenite surface to find the best adsorption site. The adsorption energy of Pb(OH)^+^ on the ilmenite surface was calculated as −16.916 kJ/mol. Figure 3 shows that all of the Pb(OH)^+^ was adsorbed on the ilmenite surface. Table 4 shows the bond population of Pb(OH)^+^.

**Table 4:** Mulliken population of adsorption configuration of Pb(OH)^+^ on the ilmenite surface.

**Pb(OH)^**+**^**	**Bond**	**Bond population**	**Bond length/Å**
I	Fe1-O1	0.22	1.921
	O1-O2	−0.06	2.520
II	Ti1-O3	0.31	2.025
	O3-O4	−0.05	2.593
III	Ti2-O5	0.22	2.000
	O6-O5	−0.04	2.686

As shown in Table 4, the atoms of Fe and Ti on the ilmenite surface interacted with O of Pb(OH)^+^ with bond populations of 0.22, 0.31, and 0.22, respectively. This confirmed that the Pb(OH)^+^ adsorbed on the ilmenite surface by covalent bonding, and that adsorption sites were abundant.

Table 5 exhibits the atom population of Fe1 and O1 before and after the adsorption of Pb(OH)^+^.

**Table 5:** Atom population of Pb(OH)^+^ on the ilmenite surface.

**Atom**	**Status**	**Total/e**	**Charge/e**
Fe1	Before adsorption	7.19	0.81
	After adsorption	7.05	0.95
O1	Before adsorption	6.66	−0.66
	After adsorption	6.58	−0.58

Known from Table 5, both Fe1 and O1 atoms have obvious electron transfer during the reaction, and 0.14 e and 0.08 e removed from Fe1 and O1 atom, respectively, which supplement the interaction between O in Pb(OH)^+^ and Fe in the ilmenite surface.

**Figure 4:**
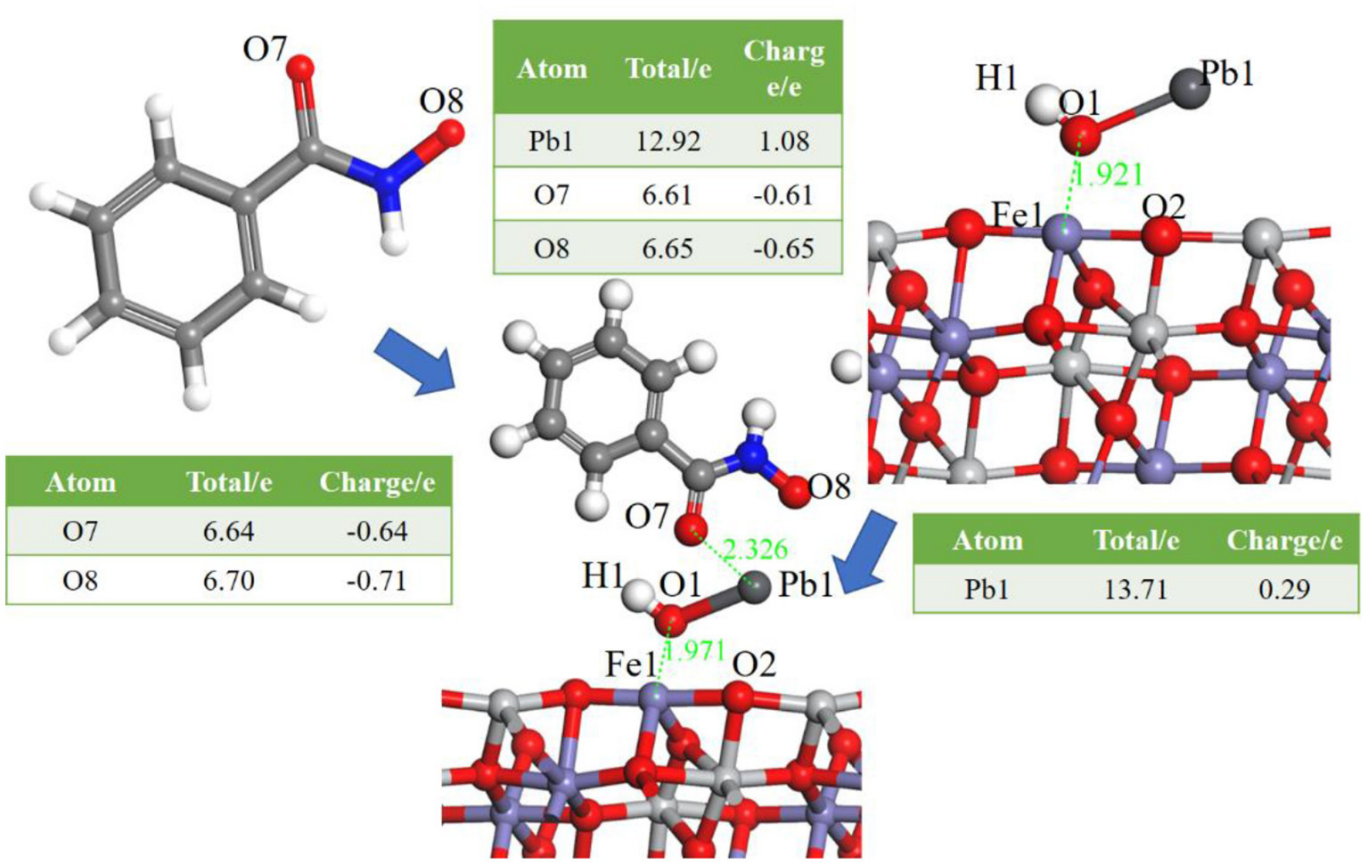
Models of BHA on the Pb(OH)^+^-functioned ilmenite surface, the Table is the atom population before and after the adsorption of BHA.

Having identified the adsorption of Pb(OH)^+^, the interaction between BHA and the ilmenite surface Pb(OH)^+^-functioned was calculated to detect the selectivity of BHA. The optimized model is shown in Figure 4.

As shown in Figure 4, BHA adsorbed on ilmenite surface via the interaction between the Pb atom of Pb(OH)^+^ and the O atom of BHA. Pb^2+^ and the O atoms formed a five-membered ring, and this interaction was stronger than that of oleate with ilmenite. The adsorption energy was determined as −101.1528 kJ/mol and the bond population analysis indicated that the BHA adsorbed on the three sites of the ilmenite surface. The five-membered ring and the abundant adsorption sites lead to the good selectivity of BHA. From the atom population analysis, the charge of Pb1 went from 0.29 to 1.08, and the two O of BHA also had obvious charge transfer which confirmed the interaction among the two O of BHA and Pb of activated ilmenite surface.

Based on the results and discussion, both oleate and BHA could adsorb on the ilmenite surface. Covalent bonds formed between Fe of ilmenite surface and the O of oleate. Due to the activation of Pb(OH)^+^, there were more adsorption sites of ilmenite for BHA (Meng et al., 2018; Ozsváth et al., 2019). And five-membered rings formed among lead species and BHA, which improved the selectivity of BHA.

## Conclusion

This study highlighted the good selectivity of BHA for ilmenite. Flotation tests were performed to discover the difference in selectivity between BHA and oleate, and the DFT calculation of the reagent-ilmenite complexes was adopted to elucidate the mechanism of the good selectivity of BHA at the atomic level.

Flotation tests showed that the selectivity of BHA was better than that of oleate. DFT calculation suggested that both oleate and BHA adsorbed on the ilmenite surface. BHA had more adsorption sites on ilmenite surfaces, as it formed five-membered rings through the O atoms of BHA and Pb^2+^ of the activator. Abundant adsorption sites and solid adsorption of five-membered rings contribute to the good selectivity of BHA.

The present investigation offers insight into the good selectivity of BHA, providing a reference for designing and screening flotation reagents for ilmenite flotation.

## Data Availability Statement

The datasets generated for this study are available on request to the corresponding author.

## Author Contributions

During investigation and paper writing, all authors participated in their assigned tasks. LL carried out flotation experiments, drafted the flotation tests, and analyzed DFT calculation results with CZ, who performed the DFT calculation and wrote the paragraphs of DFT calculation. ZY was responsible for designing the experimental scheme and outlining the manuscript structure. ZL and CL repeated flotation tests using BHA and contributed to the interpretations of flotation results and the revision of whole manuscript.

### Conflict of Interest

ZL and CL are employed by Beijing Research Institute of Chemicals Engineering and Metallurgy, CNNC. The remaining authors declare that the research was conducted in the absence of any commercial or financial relationships that could be construed as a potential conflict of interest.
